# Draft Genome Sequences of Three *Smithella* spp. Obtained from a Methanogenic Alkane-Degrading Culture and Oil Field Produced Water

**DOI:** 10.1128/genomeA.01085-14

**Published:** 2014-10-23

**Authors:** BoonFei Tan, Renata de Araújo e Silva, Trent Rozycki, Camilla Nesbø, Julia Foght

**Affiliations:** a Department of Biological Sciences, University of Alberta, Alberta, Canada; b Centro de Ciências da Saúde, Universidade Estadual do Ceará, Fortaleza, Brazil; c Centre for Ecological and Evolutionary Synthesis, University of Oslo, Oslo, Norway

## Abstract

Two draft genomes affiliated with *Smithella* spp. were obtained from a methanogenic alkane-degrading enrichment culture by single-cell sorting and metagenome contig binning, and a third was obtained by single-cell sorting of oil field produced water. Two genomes contained putative *assABC* genes encoding alkylsuccinate synthase, indicating genetic potential for fumarate activation of alkanes.

## GENOME ANNOUNCEMENT

*Smithella* and *Syntrophus* (*Syntrophaceae*) have been implicated in long-chain *n*-alkane degradation by methanogenic communities (1, 2) but few have been cultivated (2) or sequenced. The sole draft *Smithella* genome (*Smithella* ME-1; NZ_AWGX00000000.1) was coassembled from single cells sorted from a methanogenic *n*-hexadecane-degrading culture (3), but the genome of the cultivated type strain, *Smithella propionica* LYP, has not been sequenced (4). A complete *Syntrophus* genome is available (*S. aciditrophicus* SB; NC_007759.1) but it harbors no known genes for anaerobic hydrocarbon biodegradation. Thus, additional genomes from these bacterial genera would contribute to understanding hydrocarbon bioremediation under anaerobic conditions.

Single-cell sorting (http://www.bigelow.org) of a methanogenic alkane-degrading culture (SCADC) (5) and produced water from an oil field in southern Alberta (6) yielded two single cells (SC-F21 and SC-D17, respectively) affiliated with *Smithella*. These were subjected to multiple displacement amplification and sequenced as single-cell amplified genomes (SAGs) using Illumina Mi-Seq (7), and then assembled *de novo* using CLC Genomics Workbench (CLC-Bio, USA) with a k-mer size of 40. A third draft genome was obtained by binning SCADC metagenome contigs (5) using sequence homology- and composition-based methods. All genomes were subjected to sequence decontamination (7) and annotated using RAST (8).

The SC-D17 draft genome is 1.6 Mbp on 271 scaffolds with 43% GC content, whereas SC-F21 is 1.6 Mbp on 245 scaffolds with 50% GC content. The SCADC draft genome is ~3.3 Mbp on 247 scaffolds (1,000–74,000 bp) with 44% GC content. Alignment and classification of the16S rRNA gene sequence (Silva Aligner) (http://www.arb-silva.de/aligner/) indicated >98% similarity to *Smithella*, supported by phylogenetic analysis placing SC-D17, SC-F21, and SCADC within the *Smithella*-affiliated clade (2). Therefore, the two SAGs were named *Smithella* sp. SC-D17 and *Smithella* sp. SC-F21, and the SCADC binned genome was named *Smithella* sp. SCADC. Two-way average nucleotide identity analysis between *Smithella* ME-1 (NZ_AWGX00000000.1) and the three new draft genomes (1000-bp window read size) revealed high similarity to *Smithella* SC-D17 (1524 fragments; 82% similarity) and *Smithella* SCADC (2842 fragments; 86% similarity) but lower pairwise similarity to *Smithella* SC-F21 (98 fragments; 85% similarity). Comparison of single-copy gene numbers in the draft genomes to *Smithella aciditrophicus* SB (NC_007759.1) indicates that the *Smithella* SC-D17 and SC-F21 genomes are partially (>70%) complete and *Smithella* SCADC is nearly (>95%) complete.

Sequence homologs of *assA* involved in alkane activation under sulfate- and nitrate-reducing conditions by *Desulfatibacillum alkenivorans* AK-01 and *Azoarcus* sp. HxN1, respectively (9, 10), were detected in *Smithella* spp. ME-1 (3, 11), SCADC (11), and SC-D17, but not SC-F21. In *Smithella* spp. SCADC and SC-D17, *assA* is present in gene clusters containing *assB, assC*, and *masE* homologs encoding alkylsuccinate synthase subunits (9, 10). The *dsrAB* and *dsrMKJOP* genes crucial for sulfate reduction were not detected in the three draft genomes, implying the inability to reduce sulfate, as in *S. aciditrophicus* SB (12). Whole-genome comparison is under way to study the functional roles of *Smithella* spp. in methanogenic alkane degradation.

### Nucleotide sequence accession numbers.

The whole-genome shotgun projects for *Smithella* sp. SC-F21, *Smithella* sp. SC-D17, and *Smithella* sp. SCADC have been deposited at DDBJ/EMBL/GenBank under accession numbers JQIE00000000, JQOA00000000, and JQDQ00000000, respectively. The versions described in this paper are versions JQIE01000000, JQOA01000000, and JQDQ01000000.
